# Acceleration of the DNA methylation clock among lynch syndrome-associated mutation carriers

**DOI:** 10.1186/s12920-022-01183-2

**Published:** 2022-03-04

**Authors:** Marta Cuadros, Carlos Cano, Sonia Garcia-Rodriguez, José Luis Martín, Antonio Poyatos-Andujar, Francisco Ruiz-Cabello, Susana Pedrinaci, Gema Durán, Manuel Benavides, María Dolores Bautista-Ojeda, Teresa Pereda, Maria Soledad Benitez-Cantos, Pedro Medina, Armando Blanco, Antonio Gonzalez, Paul Lizardi

**Affiliations:** 1 Department of Biochemistry and Molecular Biology III and Immunology, Faculty of Medicine, University of Granada, Av. de la Investigación 11, 18007 Granada, Spain; 2 Department of Computer Science and Artificial Intelligence, University of Granada, Granada, Spain; 3 Hospital Universitario San Cecilio, Granada, Spain; 4 Hospital Virgen de Las Nieves, Granada, Spain; 5 Hospital Regional Universitario Carlos Haya, Málaga, Spain; 6 Hospital Costa del Sol, Marbella, Spain; 7 Instituto de Parasitología y Biomedicina LopezNeyra - CSIC, Granada, Spain; 8 GENYO, Centre for Genomics and Oncological Research, Pfizer/University of Granada/Andalusian Regional Government, Av. de la Ilustración 114, 18007 Granada, Spain; 9 Health Research Institute of Granada (Ibis.Granada), Av. Fuerzas Armadas 2, 18014 Granada, Spain; 10 Department of Biochemistry and Molecular Biology I, Faculty of Sciences, University of Granada, Av. de Fuente Nueva S/N, 18071 Granada, Spain

**Keywords:** DNA methylation, Lynch syndrome, Epigenetic clock

## Abstract

**Background:**

DNA methylation (DNAm) age metrics have been widely accepted as an epigenetic biomarker for biological aging and disease. The purpose of this study is to assess whether or not individuals carrying Lynch Syndrome-associated mutations are affected in their rate of biological aging, as measured by the epigenetic clock.

**Methods:**

Genome-wide bisulfite DNA sequencing data were generated using DNA from CD4 + T-cells obtained from peripheral blood using 27 patient samples from Lynch syndrome families. Horvath’s DNAm age model based on penalized linear regression was applied to estimate DNAm age from patient samples with distinct clinical and genetic characteristics to investigate cancer mutation-related aging effects.

**Results:**

Both Lynch mutation carriers and controls exhibited high variability in their estimated DNAm age, but regression analysis showed steeper slope for the Lynch mutation carriers. Remarkably, six Lynch Syndrome-associated mutation carriers showed a strong correlation to the control group, and two sisters carrying Lynch Syndrome-associated mutations, with no significant difference in lifestyle and similar chronological age, were assigned very different DNAm age.

**Conclusions:**

Future studies will be required to explore, in larger patient populations, whether specific epigenetic age acceleration is predictive of time-to-cancer development, treatment response, and survival. Epigenetic clock DNAm metrics may be affected by the presence of cancer mutations in the germline, and thus show promise of potential clinical utility for stratified surveillance strategies based on the relative risk for imminent emergence of tumor lesions in otherwise healthy Lynch Syndrome-associated mutation carriers.

## Background

The most important risk factor for chronic disease, cancer, and death is chronological age [1], and it acts as a barometer for the various biological changes that occur over the life course [2]. DNA methylation (DNAm) plays an important role in transcription control and alters consistently with age [3]. Horvath and collaborators define a DNA methylation clock (DNAm clock) as a set of CpGs whose methylation status is used with a regression algorithm to estimate the biological age of a DNA sample obtained from an individual [4]. Current views regarding the significance of epigenetic clock metrics suggest that DNAm clocks capture aging-related epigenetic modifications that are widespread and indicative of genomic, cell biology, and tissue changes that occur throughout life. These molecular changes could lead to a more precise and high-resolution knowledge of age-related disease and physiology, according to a recent review by Bell et al., 2019 [5]. Patients with HIV infection and Down syndrome have been found to have accelerated DNAm aging [3]. In addition, a large body of literature has identified DNAm as one of the key mechanisms underlying the association between aging and cancer [6, 7], showing epigenetic age acceleration might represent an early event in the development of cancerous cells and could be utilized to predict cancer risk and cancer incidence [8].

Several DNAm clocks have been proposed in the literature [4, 5]. These predictors are trained on data from different platforms and tissues, hence some variability between their predictions has been reported [5]. The clock reported by Hannum et al. [9] relied on 71 CpGs from the Illumina 450 K array, using DNA obtained from human peripheral blood samples. Horvath et al. proposed a “pan-tissue” DNAm clock [10] comprised of a subset of 353 CpGs present in the Illumina 27 k array. Recently, DNAm clocks have been proposed that are trained on age-related and disease phenotypes, such as the “PhenoAge” DNA methylation clock [4] or the “Grim Age” clock [11]. While these clocks lead to a stronger prediction on lifespan and healthspan, they are not solely based on methylation signals but require more information such as age-related biochemical measures or smoking related habits [11].

Hereditary nonpolyposis colorectal cancer (HNPCC), also known as Lynch syndrome, represents around 5% of colorectal cancers (CRC) and, it is transmitted in an autosomal dominant manner. Carriers of the different Lynch Syndrome-associated mutations in DNA mismatch repair (MMR) genes (*MLH1*, *MSH2*, *MSH6*, and *PMS2* [12]) have a significantly higher risk of developing colorectal cancer in first-degree. Moreover, these patients also have a higher risk of developing other tumor types, as endometrial adenocarcinoma, affecting at least to one female relative in 50% of Lynch syndrome pedigrees, stomach, small intestine, liver, biliary tract, brain, and ovary cancers, as well as transition cell carcinoma in ureters and renal pelvis. The average age of CRC detection in carriers of any of these mutations is in the mid 40 s to early 50 s, in contrast to the 60 years average age of onset for other sporadic CRC patients [13].

Colonoscopic and gynecological monitoring is recommended for patients with a germinal mutation in MMR genes [14, 15]. Healthy carriers of Lynch Syndrome-associated mutations are subject to a psychological burden by the knowledge that a cancer lesion is likely to emerge at any time. Physicians monitoring the health of these individuals need reliable metrics of imminent cancer risk to be able to prioritize care for the most severely affected individuals. Here we show data suggesting that DNA methylation metrics in general, and the epigenetic clock in particular, may be of utility in monitoring imminent cancer risk in healthy carriers of Lynch Syndrome-associated mutations.

## Methods

### Patients

18 Lynch syndrome patients without tumor and nine healthy relatives from 13 different families (shown in Table 1) provide peripheral blood samples for isolation of CD4 + cells. All patients with documented mutations in our study are members of large “Lynch family” cohorts. In each of these Lynch families known relatives have died from CRC caused by Lynch syndrome-associated mutations. The CRC tumors in affected family members contained germline mutations and displayed microsatellite instability, indicating that the Lynch syndrome-associated mutations are pathogenic. Samples were obtained from the Hospital Universitario San Cecilio (Granada, Spain), Hospital Virgen de las Nieves (Granada, Spain) and Hospital Regional Universitario Carlos Haya (Málaga, Spain). Participants provided written consent in accordance with the procedures of the Declaration of Helsinki and the institutional and national guidelines.

**Table 1:** Sample ID, sex, Lynch-associated mutation and age at diagnosis for the 27 participants of the 13 different families involved in the study

Sample	Sex	Mutation	Age at diagnosis	Family
HC1	Female	Negative	24	Fam1 (*MSH6*)
HC2	Male	*MSH6*	21	
HC3	Female	*MSH6*	51	Fam2 (*MSH6*)
HC4	Female	*MSH6*	52	
HC5	Female	Negative	57	
HC7	Female	Negative	50	
HC8	Male	*MSH6*	40	Fam3 (*MSH6*)
HC9	Female	*MSH6*	38	
HC11	Male	*MLH1*	43	Fam4 (*MLH1*)
VN1	Female	*MSH6*	36	Fam5 (*MSH6*)
VN2	Male	*MSH6*	40	
VN3	Female	Negative	30	
VN4	Male	Negative	39	
VN10	Female	*MSH6*	41	
VN6	Female	*MLH1*	34	Fam6 (*MLH1*)
VN8	Female	Negative	33	
VN9	Female	Negative	28	
VN7	Male	*PMS2*	29	Fam7 (*PMS2*)
M2	Female	Negative	64	Fam8
M3	Male	*MSH2*	55	Fam9 (*MSH2*)
M6	Female	Negative	34	Fam10 (*MCH1*)
M7	Male	*MCH1*	30	
M8	Male	*MLH1*	42	Fam11 (*MLH1*)
M9	Female	*MLH1*	44	
M10	Male	*MLH1*	37	Fam12 (*MLH1*)
M11	Female	*MLH1*	32	
M12	Female	*MSH2*	44	Fam13 (*MSH2*)

### Library synthesis

Peripheral blood mononuclear cells were obtained from patient blood samples by ficoll-hypaque (GE Healthcare) gradient. Dynabeads® CD4 Positive Isolation Kit (Thermo Fisher Scientific, MA, USA) was used to separate CD4 + cells. Genomic DNA was isolated from CD4 + cells using DNeasy Mini Kit (Qiagen, Carlsbad, CA). All protocols were made according to manufacturer’s procedures. Libraries were generated using the SureSelectXT Human Methyl-Seq (Agilent) Targeted Enrichment Kit (Agilent Technologies, Santa Clara, CA). Combining the SureSelectXT system and bisulfite conversion, studying 80 Mb and covering over 3.7 million CpGs. Samples were processed following the recommended Methyl‐Seq protocol Version B, January 2013. The samples target sequences were bisulfite converted using the EZ DNA Methylation‐Gold kit (Zymo Research) as described in the Methyl‐Seq protocol.

### DNA methylome sequencing

Indexed and pooled libraries were sent to the CNAG-CRG Sequencing Unit in Barcelona, Spain for DNA sequencing. Sequencing was performed on the Illumina HiSeq2000 instrument (100 bp paired‐end reads, 40 million reads per sample, representing 50X to 80X coverage). The sequencing reads were processed at CNAG-CGR in Barcelona and provided to us as fastq files.

### Computational analysis of methylation data

**Fig. 1:**
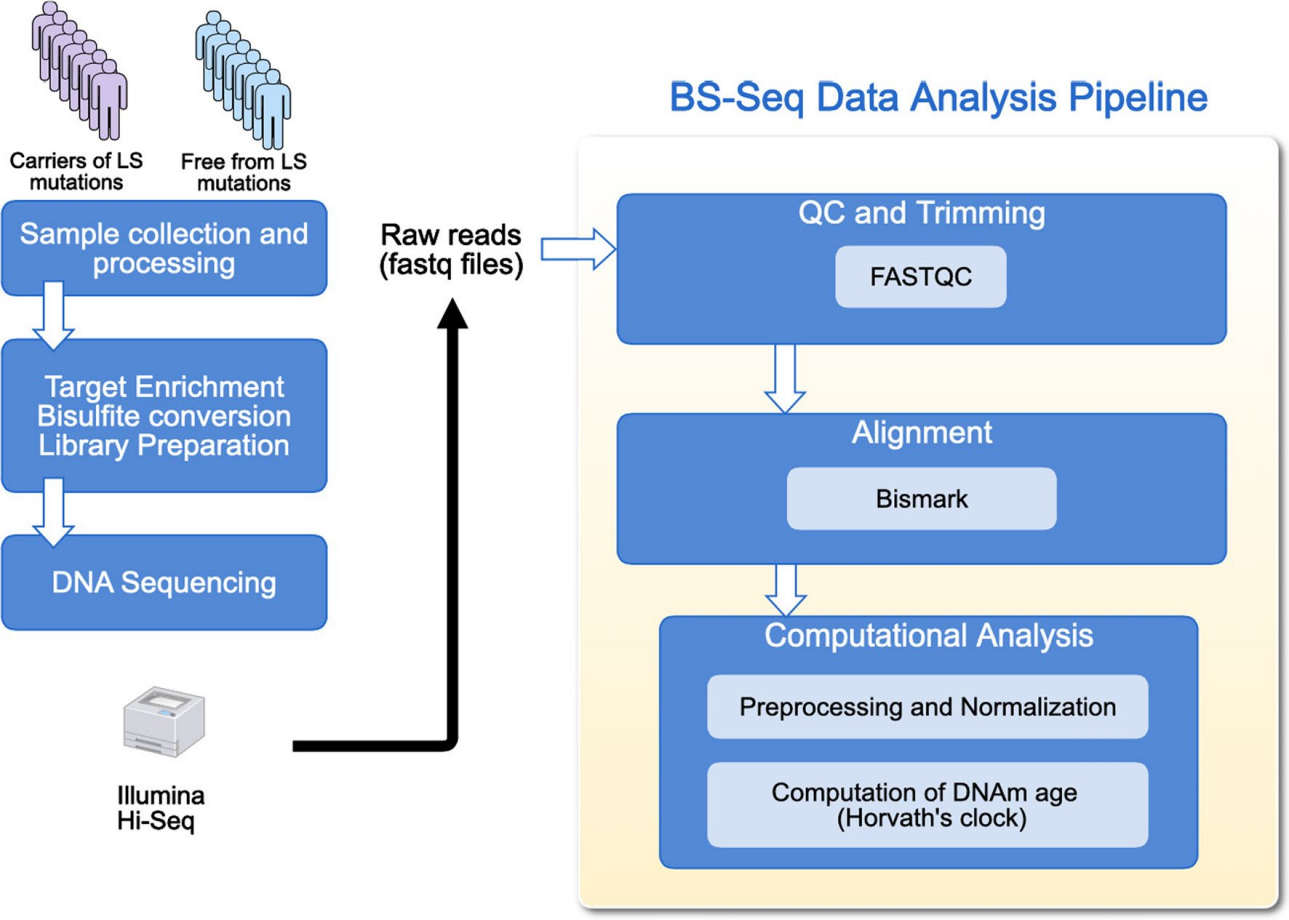
Analysis pipeline

The computational analysis of the data is depicted in Fig. 1. First, the raw reads underwent a quality control analysis with FastQC (https://github.com/s-andrews/FastQC). Second, we used Bismark [16] for BS-Seq alignment and computing the methylation ratios for all the CpG sites in the Agilent SureSelectMethylKit. Results of the alignment showed an average coverage of 61X (std ± 25) for the different samples. Horvath’s methylation age computation is based on the methylation beta values of 353 CpGs included in the Illumina450K arrays [4]. This estimation is computed as a linear combination of the beta values of these 353 CpGs using the coefficients for each CpG provided by the authors. After mapping these coordinates from Genome build 36 to Genome build 37 and use samtools to intersect Agilent SureSelectMethylKit target CpG sites with Horvath’s methylation age signature, we got a total of 317 CpG coordinates. Therefore, 36 CpGs from Horvaths’ methylation age signature could not be considered. With the remaining 317 CpGs methylation values, we computed Horvath’s methylation age using the R code distributed by the authors [4].

A linear regression was carried out separately for Lynch and Control samples using the values of age and estimated methylation with function lm in R [17].

## Results

In the present study, we compiled peripheral blood DNA methylation data, generated using target enrichment and bisulfite DNA sequencing. Target enrichment was performed using the Methyl-Seq Analysis Platform (Agilent kit, see Methods). The study cohort consisted of 18 subjects who had previously confirmed clinical and molecular diagnoses of Lynch syndrome (caused by germline mutation in one of the MMR genes, *MLH1*, *MSH2*, *MSH6*, or *PMS2*), and 9 family relatives without the mutation. All patients were healthy and had no clinical evidence of Lynch syndrome associated tumors at the date of peripheral blood extraction (Table 1).

**Fig. 2:**
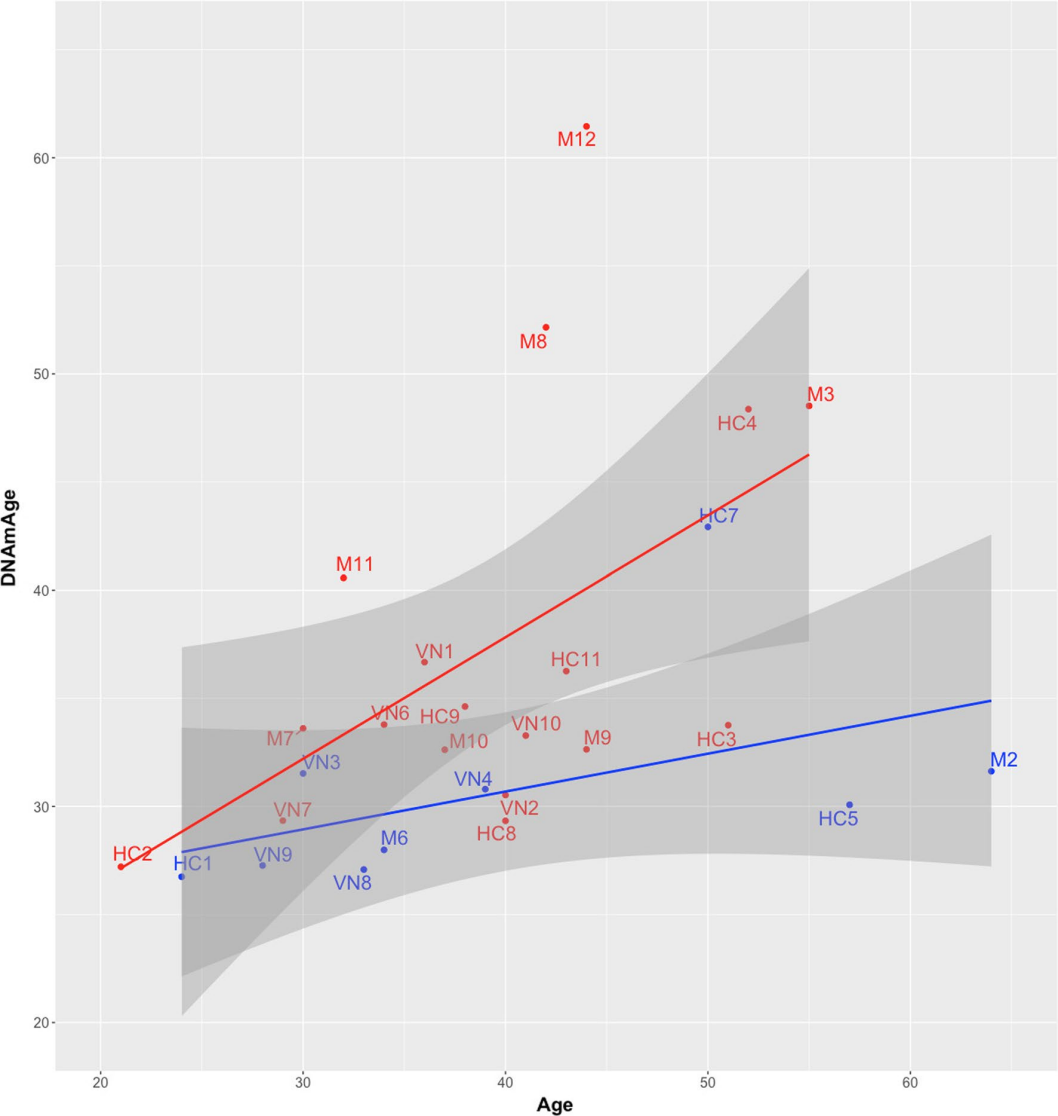
Scatterplot with the estimated methylation age (DNA*mAge*), against chronological age (*Age*) of the cases. Patients with Lynch Syndrome-associated mutations are colored in red. The nine cases negative for mutations are highlighted in blue. Regression lines for Lynch/control cases are depicted in red/blue, respectively, with 95% confidence intervals in dark grey. Multiple R^2^ value for the blue regression line is 0.23 (*p* value: 0.18). Multiple R^2^ value for the red regression line is 0.27 (*p* value: 0.026)

**Fig. 3:**
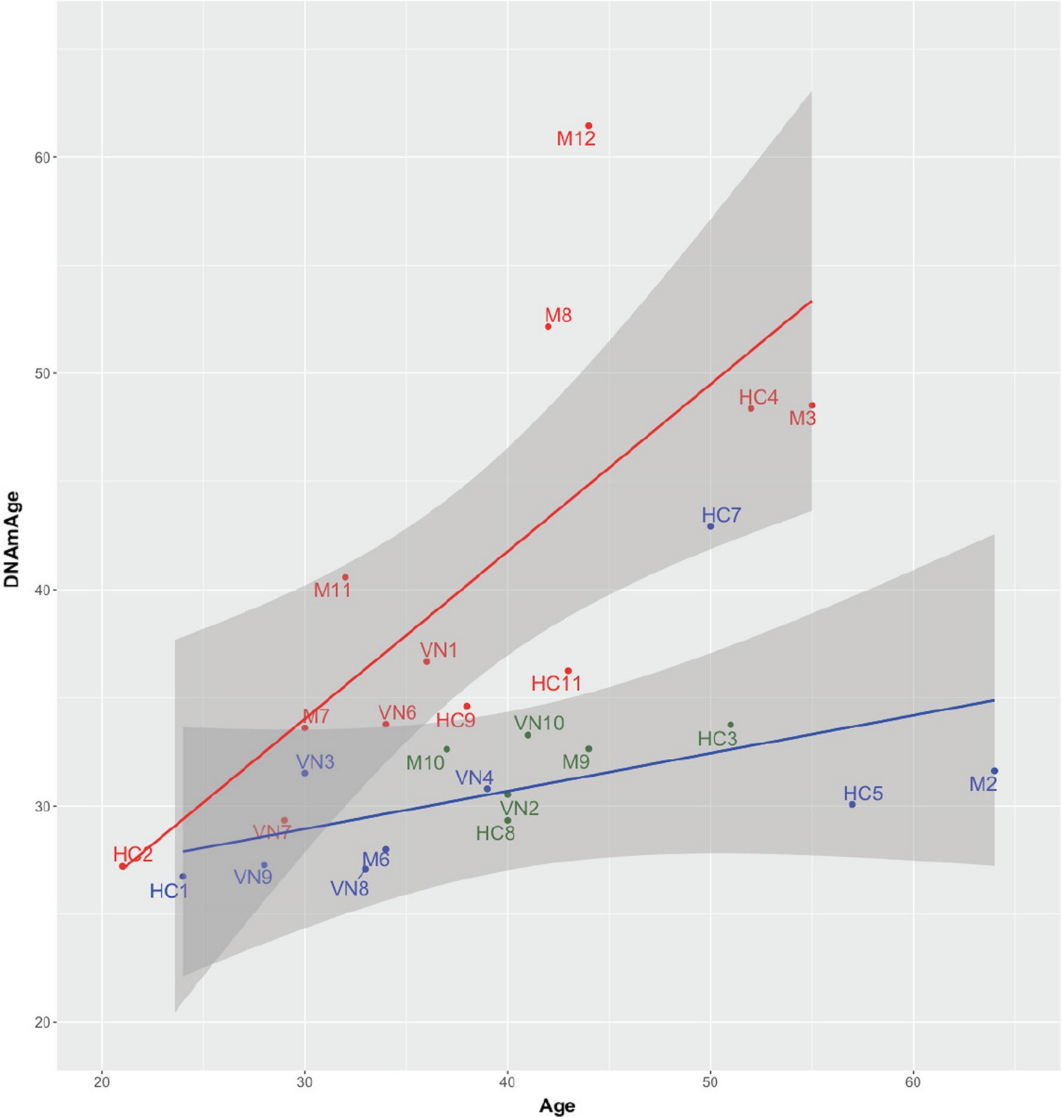
Scatterplot with the estimated methylation age (DNA*mAge*), against chronological age (*Age*) of the cases. Patients with Lynch Syndrome-associated mutations are colored in red, except for six patients (colored in green) whose metrics fall within the 95% confidence interval (grey area) of the regression line for the control cases and out of the 95% confidence interval of the regression line for Lynch cases. Cases negative for mutations are highlighted in blue. Regression lines for Lynch/control patients are depicted in red/blue, respectively, but the six Lynch cases highlighted in green are not included in the regression analysis plots. Multiple R^2^ value for the blue regression line is 0.23 (*p* value: 0.18). Multiple R^2^ value for the red regression line is 0.54 (*p* value: 0.006)

For the 27 samples, we computed the DNAm clock by Horvath et al. [4] and compared the obtained methylation age (mAge) to the chronological age. The correlation between mAge and Age (Fig. 2) was higher for the Lynch Syndrome-associated mutation carriers group (Multiple R^2^ 0.27, *p* value = 0.026) than for the control group (Multiple R^2^ 0.23, *p* value = 0.182). Both groups exhibited high variability in their estimated mAge. R^2^ values are below 30%, indicating that more variables are needed (apart from chronological age) to explain the variance of mAge. However, there are some striking cases which draw our attention in these preliminary results. One of these examples are samples HC3 and HC4, members of the same family (sisters). HC3, a carrier of a Lynch Syndrome-associated mutation does not correlate with the regression line for Lynch syndrome-associated mutation carriers, but with the regression line for control cases. The computed DNAm age for HC3 was 33% lower than the corresponding chronological age (DNAm age of 33.75 and chronological age of 51). This result is surprising, since HC4 is only 1 year older than HC3 (52 years old, at the date of the study). It is unfortunate that we do not have potentially relevant clinical data for each patient regarding differences in lifestyle (cigarette smoking, oral contraceptives, etc.). Regarding relevant clinical variables affecting the two sisters we do know the time of the endocrine disruption produced by hysterectomy. While HC3 was hysterectomized 5 years before the date of the study, HC4 was hysterectomized 20 years earlier, with a potential influence of time of surgery on epigenetic age. It is also striking that six cases which are carriers of Lynch syndrome-associated mutations (M10, HC8, VN2, VN10, M9, and HC3) have DNAm metrics that fall within the 95% confidence interval for the regression line of the nine “normal” cases that are not mutation carriers. One possible interpretation that could explain the “normal” DNAm metrics of these six cases is that in these individuals the loss of the remaining functional allele in somatic cells of the colon epithelium has not yet occurred, and microsatellite instability is not yet prevalent in epithelial cells. To further evaluate this possible interpretation, we note that among these six individuals, four are carriers of the *MSH6* mutation, for which the time of onset of CRC is known to be delayed by approximately nine years, compared to *MLH1* and *MSH2* mutation carriers [18]. The other two cases are carriers of the *MLH1* mutation, and are 37 and 44 years old, respectively. The data in the Ryan et al. study [18] showed that approximately 50% of *MLH1* mutation carriers develop CRC tumors after the age of 46, therefore it is possible that DNAm metrics for these two individuals reflect their excellent health status, mediated by a delay in the onset of mismatch repair defects and microsatellite instability. In Fig. 3 we show a modified DNAm scatter plot of all 27 cases, in which we have excluded the six mutation-carrying outliers (highlighted in green) from the linear regression analysis of the Lynch mutation carriers. The slope of the regression line for the 12 remaining mutation carriers (highlighted in red) is now steeper, possibly reflecting the deteriorating health status and acceleration of the epigenetic clock in the subset of mutation carriers for whom microsatellite instability and a high mutational burden are already prevalent in colonic tissues.

## Discussion

The data obtained in this study shows that the majority of individuals carrying Lynch syndrome-associated mutations display an acceleration of the epigenetic clock, as defined by the Horvath CpG methylation metrics. However, six out of 18 individuals with genetically documented mutations did not show acceleration of the epigenetic clock, suggesting that for these cases there has been a delay in the onset of the Lynch syndrome disease process.

Physicians monitoring the health of individuals known to be carriers of Lynch mutations need reliable metrics of relative cancer risk to be able to assess and prioritize care according to the likelihood of the imminent appearance of a cancer lesion. Here we show data indicating that DNA methylation metrics may be able to stratify carriers and non-carriers on the basis of the rate of biological aging as defined by the epigenetic clock. With follow-up studies involving a larger number of subjects, it may be possible to refine these biomarkers of the biological aging process, so they can be of utility in monitoring imminent cancer risk in healthy carriers of Lynch Syndrome-associated mutations. When fully developed, these DNAm biomarkers could be of potential clinical utility for stratified surveillance strategies that will facilitate cancer prevention in Lynch syndrome patients. Prospective studies, where the biological age based on the epigenetic clock is measured every year until cancers actually appear in certain individuals have potential to reveal additional correlations or insights, such as the possibility of accelerated biological aging just before the time of tumor emergence.

We note that our study has certain limitations relating to statistical significance, due to the small sample size of the patient population. In addition, the clinical annotation of the subjects who participated in the study suffers from lack of data relating to nutrition habits and lifestyle choices, such as contraceptives, alcohol consumption, smoking, etc. These limitations could be addressed by performing studies with larger numbers of Lynch syndrome patients, and also by collecting samples from the same individuals every year in order to generate longitudinal data sets.

## Conclusions

Epigenetic clock DNAm metrics may be affected by the presence of cancer mutations in the germline, and thus show promise of potential clinical utility for assessment of relative risk related to imminent emergence of tumor lesions in otherwise healthy Lynch Syndrome-associated mutation carriers.

## Data Availability

Bisulfite-Seq data are available at NCBI BioProject and Sequence Read Archive with Accession Number: PRJNA804259. http://www.ncbi.nlm.nih.gov/bioproject/804259, https://www.ncbi.nlm.nih.gov/Traces/study/?acc=PRJNA804259.

## Abbreviations

DNAm: DNA methylation
DNAm clock: DNA methylation clock
HNPCC: Hereditary nonpolyposis colorectal cancer
CCR: Colorectal cancer
MMR: Mismatch repair
